# *In vitro* activity of ceftazidime/avibactam against clinical isolates of Enterobacterales and *Pseudomonas aeruginosa* from Middle Eastern and African countries: ATLAS global surveillance programme 2015–18

**DOI:** 10.1093/jacamr/dlab067

**Published:** 2021-06-01

**Authors:** James A Karlowsky, Samuel K Bouchillon, Ramy El Mahdy Kotb, Naglaa Mohamed, Gregory G Stone, Daniel F Sahm

**Affiliations:** 1 IHMA, Schaumburg, IL, USA; 2 Department of Medical Microbiology and Infectious Diseases, Max Rady College of Medicine, University of Manitoba, Winnipeg, Manitoba, Canada; 3 Pfizer Inc, Dubai, UAE; 4 Pfizer Inc, New York, NY, USA; 5 Pfizer Inc, Groton, CT, USA

## Abstract

**Objectives:**

To assess the *in vitro* activity of ceftazidime/avibactam against a recent, 2015–18, collection of clinical isolates of Gram-negative bacilli from Middle Eastern and African countries with a focus on isolates from ICUs and with MDR and difficult-to-treat resistance (DTR) phenotypes.

**Methods:**

Antimicrobial susceptibility testing of 4608 isolates of Enterobacterales (997 isolates from ICU patients) and 1358 isolates of *Pseudomonas aeruginosa* (374 isolates from ICU patients) was performed by CLSI broth microdilution methodology in a central laboratory. MICs were interpreted using both CLSI (2020) and EUCAST (2020) MIC breakpoints.

**Results:**

Most isolates of Enterobacterales (Middle East: ICU, 99.1% susceptible, non-ICU, 99.1%; Africa: ICU, 96.9% susceptible, non-ICU, 98.3%) and *P. aeruginosa* (Middle East: ICU, 93.4%, non-ICU, 92.1%; Africa: ICU, 89.8%; non-ICU, 94.1%) were susceptible to ceftazidime/avibactam. Applying CLSI and EUCAST breakpoints, MDR rates were similar for Enterobacterales (27.8%–36.0% of isolates) and *P. aeruginosa* (25.0%–36.4%) while DTR rates were lower for Enterobacterales (1.6%–1.8%) than for *P. aeruginosa* (5.2%–7.4%). Percentage susceptible rates for ceftazidime/avibactam for MDR Enterobacterales were 96.8%–97.5% (Middle East) and 92.5%–94.3% (Africa) while rates for *P. aeruginosa* were 70.1%–80.0% (Middle East) and 69.5%–78.2% (Africa). 60.5%–65.8% (Middle East) and 38.9%–52.2% (Africa) of isolates of Enterobacterales with DTR phenotypes were ceftazidime/avibactam susceptible as were 29.2%–31.1% (Middle East) and 28.2%–35.8% (Africa) of DTR *P. aeruginosa*.

**Conclusions:**

Overall, the isolates of Enterobacterales and *P. aeruginosa* tested from Middle Eastern and African countries were highly susceptible to ceftazidime/avibactam. Most MDR and many DTR isolates of Enterobacterales and *P. aeruginosa* were susceptible to ceftazidime/avibactam.

## Introduction

It is important to identify clinical isolates of Gram-negative bacilli (GNB) with resistance determinants and MDR phenotypes that limit empirical and first-line therapeutic options, particularly in ICUs. MDR is frequently defined using criteria established by Magiorakos *et al*.,^1^ that is, isolates non-susceptible (intermediate or resistant) to at least one agent in three or more antimicrobial categories. More recently, Kadri *et al*.^2^ identified a more stringent phenotypic category termed difficult-to-treat resistance (DTR) that focuses on treatment-limiting non-susceptibility (intermediate or resistant) to all first-line agents (all β-lactams, including carbapenems, and fluoroquinolones). DTR has been associated with increased patient mortality/treatment failure and requires clinicians to use other potentially less effective or more toxic agents such as aminoglycosides, tigecycline and polymyxins.^2^

To date, only two surveillance studies have published region-specific data describing GNB isolates from Middle Eastern and African countries tested against ceftazidime/avibactam.^3^^,^^4^ Both of these studies grouped Middle Eastern and African countries together and did not provide information describing the activity of ceftazidime/avibactam against ICU isolates or isolates with MDR or DTR phenotypes. Other publications from Middle Eastern and African countries describing the activity of ceftazidime/avibactam against GNB isolates only include case reports (seven cases in total).^5^^,^^6^ One of these reports, a case series from a tertiary-care centre in Saudi Arabia, reported that five of six patients infected with carbapenem-resistant Enterobacterales or *Pseudomonas aeruginosa* achieved both clinical and microbiological cure when treated with ceftazidime/avibactam.^5^ The current study intended to evaluate the *in vitro* activity of ceftazidime/avibactam against Enterobacterales and *P. aeruginosa* isolates, gathered in 2015–18, from Middle Eastern and African countries, with a focus on ICU and non-ICU patient isolates with MDR and DTR phenotypes to assess its potential benefit against these resistant isolate subsets.

## Materials and methods

### Bacterial isolates

Bacterial isolates tested in the current study were collected as a part of the ATLAS global surveillance programme by laboratories in 12 medical centres in four Middle Eastern countries (six in Israel, two in Jordan, three in Kuwait, one in Saudi Arabia) and 13 medical centres in three countries in Africa (four in Morocco, three in Nigeria, six in South Africa) from 2015 to 2018. Isolates were from bloodstream, intra-abdominal, respiratory tract, skin and soft tissue and urinary tract infection specimen sources and comprised 4608 isolates of Enterobacterales (997 ICU isolates, 3611 non-ICU isolates) and 1358 isolates of *P. aeruginosa* (374 ICU isolates, 984 non-ICU isolates) (Table S1, available as Supplementary data at *JAC-AMR* Online). In Middle Eastern countries, ICU isolates were contributed by medical ICUs (45%, 330/737), paediatric ICUs (37%, 273/737), unspecified ICUs (9%, 70/737) and surgical ICUs (9%, 64/737). In African countries, ICU isolates were submitted by unspecified ICUs (41% 259/634), medical ICUs (30% 190/634), paediatric ICUs (15%, 97/634) and surgical ICUs (14%, 88/634). All isolates were shipped to IHMA (Schaumburg, IL, USA) where their identities were confirmed using MALDI-TOF MS (Bruker Daltonics, Billerica, MA, USA).

### Antimicrobial susceptibility testing

Antimicrobial susceptibility testing was performed by IHMA using CLSI broth microdilution methodology.^7^^,^^8^ MICs were interpreted using CLSI^8^ and EUCAST^9^ MIC breakpoints. EUCAST MIC breakpoints listed as ‘susceptible, increased exposure’ (specifically species of Enterobacterales [*Morganella morganii*, *Proteus* spp. and *Providencia* spp.] tested against imipenem; *P. aeruginosa* tested against cefepime, ceftazidime, imipenem, levofloxacin and piperacillin/tazobactam) were considered susceptible when reporting individual agent, MDR and DTR results.^9^ MDR and DTR phenotypes were identified using the criteria of Magiorakos *et al*.^1^ and Kadri *et al*.,^2^ respectively (Table S2).

### Statistical analysis

The χ^2^ statistic with Yates correction (XLSTAT version 2019.1.3) was used to establish statistical significance (*P < *0.05) between categorical variables.

### Ethics

Ethical approval was not required.

## Results

Isolates of Enterobacterales from Middle Eastern (99.1% susceptible) and African (98.0% susceptible) countries were highly susceptible to ceftazidime/avibactam; 68%–72% of isolates of Enterobacterales were susceptible to ceftazidime alone (Table 1). The susceptibilities of isolates of *P. aeruginosa* from Middle Eastern and African countries were highest for ceftazidime/avibactam (92%–93% susceptible) and amikacin (92%–93% susceptible); 81%–84% of isolates of *P. aeruginosa* were susceptible to ceftazidime alone.

**Table 1.** *In vitro* susceptibility of isolates of Enterobacterales and *P. aeruginosa* from Middle Eastern and African countries to 11 antimicrobial agents with MICs interpreted by CLSI and EUCAST breakpoints

Geographic region/ Bacterial group/species^b^	*n*	Percentage susceptible; MICs interpreted by CLSI/EUCAST^a^ breakpoints										
		AMK	ATM	CAZ	CZA	CST	CRO^c^	FEP	IPM	MEM	LVX	TZP
Middle East												
Enterobacterales (all)^d^	2757	98.0/95.4	71.8/67.6	72.1/67.6	99.1/99.1	0/82.3	67.9/67.9 (1494)	72.8/70.9	86.1/95.3	97.9/98.1	64.3/64.3	87.6/81.8
* Citrobacter* spp. (all)	239	99.2/99.2	88.3/84.9	87.0/85.4	100/100	0/99.6	86.9/86.9 (168)	96.7/95.0	97.1/99.6	99.6/99.6	83.3/83.3	94.6/88.7
* Citrobacter freundii*	117	100/100	79.5/76.9	76.9/74.4	100/100	0/100	78.7/78.7 (89)	94.0/91.5	94.9/100	99.1/99.1	70.9/70.9	88.9/84.6
* Citrobacter koseri*	104	98.1/98.1	97.1/97.1	97.1/97.1	100/100	0/99.0	97.1/97.1 (68)	99.0/98.1	99.0/99.0	100/100	97.1/97.1	100/92.3
* Enterobacter* spp. (all)	250	99.6/98.4	71.6/67.6	70.0/67.2	97.6/97.6	0/85.6	62.4/62.4 (117)	82.4/77.6	86.4/96.0	96.4/96.4	77.2/77.2	78.0/72.8
* Enterobacter cloacae*	220	99.5/98.2	69.1/65.5	67.3/64.5	97.3/97.3	0/90.9	58.5/58.5 (106)	80.5/75.0	91.4/95.5	95.9/95.9	75.5/75.5	76.4/70.5
* E. coli*	770	98.4/94.4	65.1/60.4	68.7/61.3	99.9/99.9	0/99.2	63.0/63.0 (416)	64.8/62.5	99.0/99.6	99.9/99.9	53.5/53.5	91.8/86.6
* Klebsiella aerogenes*	130	98.5/97.7	77.7/72.3	73.1/71.5	99.2/99.2	0/100	72.9/72.9 (59)	95.4/93.8	63.8/98.5	98.5/98.5	95.4/95.4	80.8/74.6
* Klebsiella oxytoca*	130	100/100	93.8/90.0	97.7/96.9	100/100	0/99.2	89.5/89.5 (76)	96.2/95.4	100/100	100/100	93.1/93.1	94.6/93.8
* Klebsiella pneumoniae*	794	96.0/93.2	55.3/53.0	55.0/52.8	98.5/98.5	0/98.1	55.8/55.8 (423)	55.8/54.4	94.3/95.7	94.8/95.3	59.9/59.9	78.1/68.6
* M. morganii*	76	100/96.1	93.4/85.5	81.6/72.4	100/100	0/0	91.7/91.7 (36)	97.4/97.4	5.3/46.1	100/100	28.9/28.9	98.7/97.4
* Proteus* spp. (all)	199	100/96.5	96.5/94.0	98.0/95.0	100/100	0/0	76.6/76.6 (111)	81.9/81.4	32.7/84.4	100/100	57.3/57.3	100/97.5
* Proteus mirabilis*	127	100/95.3	95.3/92.1	96.9/93.7	100/100	0/0	74.3/74.3 (70)	73.2/72.4	32.3/84.3	100/100	39.4/39.4	100/96.1
* Proteus vulgaris* ^b^	56	100/98.2	100/98.2	100/96.4	100/100	0/0	85.2/85.2 (27)	98.2/98.2	32.1/80.4	100/100	89.3/89.3	100/100
* Providencia* spp.^e^	71	90.1/85.9	93.0/69.0	87.3/57.7	93.0/93.0	0/0	64.0/64.0 (50)	60.6/60.6	59.2/93.0	95.8/95.8	26.8/26.8	95.8/94.4
* S. marcescens*	87	100/98.9	98.9/97.7	98.9/98.9	100/100	0/4.6	96.9/96.9 (32)	100/98.9	90.8/97.7	100/100	94.3/94.3	98.9/96.6
* P. aeruginosa*	827	92.7/92.7	67.8/79.7	80.5/80.5	92.4/92.4	0/99.5	NA/NA (440)	81.0/81.0	66.0/73.0	72.9/72.9	62.8/62.8	74.8/74.8
Africa												
Enterobacterales (all)^f^	1851	97.8/96.3	71.6/69.5	71.7/69.0	98.0/98.0	0/84.1	72.7/72.7 (856)	71.9/71.0	85.0/93.1	95.8/97.0	67.5/67.5	84.3/79.4
* Citrobacter* spp. (all)	95	98.9/98.9	84.2/84.2	84.2/82.1	97.9/97.9	0/100	87.0/87.0 (54)	90.5/89.5	92.6/95.8	95.8/97.9	87.4/87.4	89.5/83.2
* C. freundii*	42	97.6/97.6	73.8/73.8	73.8/71.4	95.2/95.2	0/100	79.2/79.2 (24)	88.1/85.7	85.7/90.5	90.5/95.2	83.3/83.3	78.6/73.8
* C. koseri*	42	100/100	92.9/92.9	92.9/90.5	100/100	0/100	91.7/91.7 (24)	92.9/92.9	100/100	100/100	90.5/90.5	97.6/90.5
* Enterobacter* spp. (all)	223	97.8/96.4	69.5/67.7	70.0/65.9	96.9/96.9	0/91.9	79.4/79.4 (63)	72.2/70.4	83.0/91.9	92.8/95.1	81.2/81.2	78.5/76.7
* E. cloacae*	187	97.3/95.7	64.7/63.6	65.2/61.5	96.3/96.3	0/95.7	76.8/76.8 (56)	67.9/65.8	84.5/90.4	91.4/94.1	78.1/78.1	74.9/72.7
* E. coli*	575	99.7/97.4	79.1/77.0	81/77.7	100/100	0/99.7	82.8/82.8 (267)	79.0/78.3	99.1/99.7	99.7/100	57.2/57.2	93.0/90.3
* K. aerogenes*	50	100/100	94.0/92.0	88.0/86.0	100/100	0/98.0	89.3/89.3 (28)	100/100	82.0/100	100/100	100/100	92.0/90.0
* K. oxytoca*	56	94.6/87.5	89.3/87.5	92.9/92.9	100/100	0/100	82.9/82.9 (35)	91.1/89.3	100/100	100/100	92.9/92.9	89.3/89.3
* K. pneumoniae*	547	96.3/95.4	46.3/44.1	46.6/44.2	96.0/96.0	0/99.5	45.8/45.8 (273)	45.0/44.4	88.8/92.9	91.2/93.2	58.5/58.5	69.1/58.0
* Klebsiella variicola*	30	100/100	86.7/86.7	90.0/86.7	100/100	0/100	85.0/85.0 (20)	86.7/86.7	100/100	100/100	90.0/90.0	96.7/96.7
* M. morganii*	49	100/98.0	93.9/91.8	85.7/81.6	98.0/98.0	0/0	94.7/94.7 (19)	98.0/98.0	2.0/34.7	98.0/98.0	57.1/57.1	93.9/89.8
* Proteus* spp. (all)	126	96.8/96.0	96.0/94.4	96.0/95.2	100/100	0/0	90.5/90.5 (63)	95.2/94.4	38.1/82.5	100/100	83.3/83.3	99.2/99.2
* P. mirabilis*	87	97.7/96.6	95.4/93.1	95.4/94.3	100/100	0/0	95.2/95.2 (42)	94.3/93.1	34.5/82.8	100/100	75.9/75.9	98.9/98.9
* P. vulgaris*	31	93.5/93.5	96.8/96.8	96.8/96.8	100/100	0/0	82.4/82.4 (17)	96.8/96.8	51.6/80.6	100/100	100/100	100/100
* Providencia* spp.^g^	37	94.6/94.6	97.3/83.8	75.7/75.7	89.2/89.2	0/0	93.3/93.3 (15)	89.2/86.5	35.1/81.1	89.2/91.9	59.5/59.5	89.2/86.5
* S. marcescens*	60	96.7/95.0	90.0/90.0	90.0/86.7	98.3/98.3	0/3.3	100/100 (18)	91.7/90.0	86.7/95.0	95.0/96.7	85.0/85.0	93.3/93.3
* P. aeruginosa*	531	91.5/91.5	69.3/85.9	83.6/83.6	92.7/92.7	0/99.8	NA/NA (273)	79.7/79.7	70.4/75.7	75.5/75.5	66.5/66.5	75.7/75.7

AMK, amikacin; ATM, aztreonam; CAZ, ceftazidime; CZA, ceftazidime/avibactam; CST, colistin; CRO, ceftriaxone; FEP, cefepime; IPM, imipenem; MEM, meropenem; LVX, levofloxacin; TZP, piperacillin/tazobactam; NA, not available.

a Isolates of Enterobacterales (specifically, *M. morganii*, *Proteus* spp. and *Providencia* spp. tested against imipenem) and *P. aeruginosa* (tested against aztreonam, ceftazidime, cefepime, imipenem, levofloxacin and piperacillin/tazobactam) interpreted as susceptible by EUCAST MIC breakpoints included isolates testing in the susceptible, increased exposure category.

b Species with fewer than 30 isolates are not shown individually.

c CRO, ceftriaxone is shown with the number of isolates of Enterobacterales tested in brackets (*n*) as ceftriaxone was not tested against all isolates of Enterobacterales.

d The 18 isolates of *Citrobacter* that were undefined in the table were: 3 *Citrobacter amalonauticus*, 6 *Citrobacter braakii*, 1 *Citrobacter farmer*, 1 *Citrobacter gillenii*, 1 *Citrobacter murliniae* and 6 *Citrobacter sedlakii*. The 30 isolates of *Enterobacter* that were undefined in the table were: 15 *Enterobacter asburiae*, 4 *Enterobacter kobei* and 11 *Enterobacter* sp. The 16 isolates of *Proteus* that were undefined in the table were: 13 *Proteus hauseri*, 2 *Proteus penneri* and 1 *Proteus* sp. The 11 other isolates undefined in the table were: 7 *K. variicola*, 2 *Raoultella ornithinolytica*, 1 *Salmonella* sp. and 1 *Serratia* sp.

e The 71 isolates of *Providencia* spp. were composed of 1 *Providencia alcalifaciens*, 14 *Providencia rettgeri* and 56 *P. stuartii*.

f The 11 isolates of *Citrobacter* that were undefined in the table were: 4 *C. amalonauticus* and 7 *C. braakii*. The 36 isolates of *Enterobacter* that were undefined in the table were: 15 *E. asburiae*, 7 *E. kobei*, 41 *Enterobacter ludwigii* and 13 *Enterobacter* sp. The 8 isolates of *Proteus* that were undefined in the table were: 6 *P. hauseri* and 2 *P. penneri*. The 3 other isolates undefined in the table were: 1 *Pantoea dispersa*, 1 *R. ornithinolytica* and 1 *Serratia liquefaciens*.

g The 37 isolates of *Providencia* spp. were composed of 16 *P. rettgeri* and 21 *P. stuartii*.

Percentages of isolates of individual species from ICU and non-ICU patients were largely similar between sites in the Middle East and Africa (Table S1). However, ICU and non-ICU isolates of individual species of Enterobacterales and *P. aeruginosa* from Middle Eastern and African countries demonstrated significant differences in percentage susceptibility for agents other than ceftazidime/avibactam by both CLSI or EUCAST breakpoints (Tables S3 and S4).

CLSI breakpoints identified more isolates of Enterobacterales and *P. aeruginosa* as MDR and DTR than EUCAST breakpoints with the notable exception of *P. aeruginosa* from the Middle East for which both CLSI and EUCAST breakpoints identified 38 isolates as DTR (Table 2). Ceftazidime/avibactam inhibited most isolates (92.5%–97.5%) of MDR Enterobacterales and 69.5% to 80.0% of MDR *P. aeruginosa* from Middle Eastern and African countries at its susceptible MIC breakpoint (MIC ≤8 mg/L). Many isolates of DTR Enterobacterales (38.9%–65.8%) and DTR *P. aeruginosa* (28.2%–35.8%) were also susceptible to ceftazidime/avibactam.

**Table 2.** *In vitro* susceptibility of Enterobacterales and *P. aeruginosa* with MDR and DTR phenotypes defined by CLSI and EUCAST MIC breakpoints stratified by geographic region (Middle East, Africa)

Geographic region/ Bacterial group/ species	Percentage susceptible (CLSI MIC breakpoints^a^)											Percentage susceptible (EUCAST MIC breakpoints^b^)										
	MDR						DTR					MDR						DTR				
	*n*	CZA	FEP	MEM	TZP		*n*	CZA	FEP	MEM	TZP	*n*	CZA	FEP	MEM	TZP		*n*	CZA	FEP	MEM	TZP
Middle East																						
Enterobacterales	1015	97.5	27.2	94.4	69.0		38	65.8	0	0	0	788	96.8	17.5	72.0	48.1		38	60.5	0	0	0
* P. aeruginosa*	315	80.0	50.2	40.4	35.6		61	31.1	0	0	0	211	70.1	19.0	18.0	7.0		48	29.2	0	0	0
Africa																						
Enterobacterales	646	94.3	20.7	87.9	58.2		46	52.2	0	0	0	494	92.5	9.4	64.6	37.7		36	38.9	0	0	0
* P. aeruginosa*	179	78.2	40.8	36.9	29.1		39	28.2	0	0	0	128	69.5	15.6	14.1	5.5		23	35.8	0	0	0

CZA, ceftazidime/avibactam; FEP, cefepime; MEM, meropenem; TZP, piperacillin/tazobactam.

a Using CLSI MIC breakpoints, 36.8% (1015/2757) of Enterobacterales and 38.1% (315/827) of *P. aeruginosa* were MDR in Middle Eastern countries and 34.9% (646/1851) of Enterobacterales and 33.7% (179/531) of *P. aeruginosa* were MDR in African countries; 1.4% (38/2757) of Enterobacterales and 7.4% (61/827) of *P. aeruginosa* were MDR in Middle Eastern countries and 2.5% (46/1851) of Enterobacterales and 7.3% (39/531) of *P. aeruginosa* were MDR in African countries.

b Using EUCAST MIC breakpoints, 28.6% (788/2757) of Enterobacterales and 25.5% (211/827) of *P. aeruginosa* were MDR in Middle Eastern countries and 26.7% (494/1851) of Enterobacterales and 24.1% (128/531) of *P. aeruginosa* were MDR in African countries; 1.4% (38/2757) of Enterobacterales and 5.8% (48/827) of *P. aeruginosa* were MDR in Middle Eastern countries and 1.9% (36/1851) of Enterobacterales and 4.3% (23/531) of *P. aeruginosa* were MDR in African countries.

Using CLSI MIC breakpoints, MDR rates among species of GNB from both Middle Eastern and African countries combined ranged from 8.2% for *Serratia marcescens* to 63.2% for *M. morganii* and DTR rates ranged from 0.1% for *Escherichia coli* to 7.4% for *P. aeruginosa* (Figure S1). Using EUCAST MIC breakpoints, MDR rates ranged from 7.5% for *S. marcescens* to 44.2% for *Providencia stuartii* and DTR rates ranged from 0.1% for *E. coli* to 5.2% for *P. aeruginosa*. For all isolates of Enterobacterales, rates of MDR were 20 times greater than DTR using CLSI MIC breakpoints and 17 times greater than DTR using EUCAST MIC breakpoints. For *P. aeruginosa*, rates of MDR were 5 times greater than DTR using both CLSI and EUCAST MIC breakpoints.

For Enterobacterales, using CLSI MIC breakpoints, MDR phenotypes were 14 times more common than DTR phenotypes in ICU isolates and 23 times more common in non-ICU isolates (Figure S2). For Enterobacterales, using EUCAST MIC breakpoints, MDR phenotypes were 13 times more common than DTR phenotypes in ICU isolates and 19 times more common in non-ICU isolates. For Enterobacterales both MDR phenotypes and DTR phenotypes were significantly more common (*P < *0.05) for ICU than non-ICU isolates using both CLSI and EUCAST MIC breakpoints. For *P. aeruginosa*, MDR phenotypes were 5 times more common than DTR phenotypes in both ICU and non-ICU isolates using both CLSI and EUCAST MIC breakpoints. For *P. aeruginosa*, the differences in percentage of MDR or DTR phenotypes among ICU and non-ICU isolates were not significant (*P > *0.05) using either CLSI or EUCAST MIC breakpoints.

For Enterobacterales, using CLSI MIC breakpoints, MDR phenotypes were 12 times (blood) to 33 times (urinary tract) more common than DTR phenotypes (Figure S3). For Enterobacterales, using EUCAST MIC breakpoints, MDR phenotypes were 11 times (blood) to 25 times (urinary tract) more common than DTR phenotypes. For Enterobacterales, the percentage of isolates with MDR phenotypes and DTR phenotypes were both significantly different (*P < *0.05) among specimen sources using both CLSI and EUCAST MIC breakpoints. Blood isolates had the highest percentage of isolates with both MDR and DTR phenotypes (*P < *0.05). For *P. aeruginosa*, MDR phenotypes were 4 to 8 times more common than DTR phenotypes across the five specimen sources using both CLSI and EUCAST MIC breakpoints. Differences in the percentage of MDR or DTR phenotypes of *P. aeruginosa* isolates across the five specimen sources were not significantly different (*P > *0.05) using either CLSI or EUCAST MIC breakpoints. For *P. aeruginosa*, blood isolates had the lowest percentage of isolates that were MDR and DTR.

## Discussion

The current study determined that most isolates of Enterobacterales from study centres in Middle Eastern (ICU, 99.1% susceptible; non-ICU, 99.1%) and African (ICU, 96.9% susceptible; non-ICU, 98.3%) countries and *P. aeruginosa* from Middle Eastern (ICU, 93.4%; non-ICU, 92.1%) and African (ICU, 89.8%; non-ICU, 94.1%) countries were susceptible to ceftazidime/avibactam (MIC ≤8 mg/L) (Table 1). Of the agents tested, only ceftazidime/avibactam and amikacin demonstrated susceptibility rates approaching 100% (95.1%–99.1%) for Enterobacterales from both ICU and non-ICU isolates from both Middle Eastern and African countries when MICs were interpreted by either CLSI or EUCAST MIC breakpoints. Ceftazidime/avibactam and amikacin were also the most active agents tested against *P. aeruginosa* for both ICU and non-ICU isolates from both Middle Eastern and African countries when MICs were interpreted by either CLSI or EUCAST MIC breakpoints (89.8%–96.5% susceptible).

Using CLSI or EUCAST MIC breakpoints, MDR rates were up to 250 times higher than the corresponding DTR rates for the same collections of isolates (e.g. *E. coli*, Figure S1). This observation suggests that many MDR phenotypes identified for Enterobacterales include antimicrobial agents not considered first-line agents (i.e. β-lactams and fluoroquinolones) and may be of less importance in terms of impact on patient care, treatment options or public health. Published studies describing DTR isolates are currently limited and describe primarily bacteraemia isolates.^2^^,^^10–12^ Rates of DTR have ranged from <1% to 1.4% for Enterobacterales and from 2.3% to 9.0% *P. aeruginosa* in studies published by investigators in the United States, Italy and Korea,^2^^,^^10–12^ and are comparable with the rates observed in the current study.

Avibactam, a non-β-lactam diazabicyclooctane inhibitor of Ambler class A β-lactamases, including ESBLs and KPCs, class C (AmpC) β-lactamases and some class D (OXA-48) β-lactamases, restores activity to ceftazidime in most isolates of Enterobacterales and *P. aeruginosa* that carry these β-lactamases.^3^^,^^13–15^ Ceftazidime/avibactam also inhibits clinical isolates of *P. aeruginosa* that are carbapenem resistant because of a combination of porin loss or upregulated antimicrobial agent efflux and elevated production of *Pseudomonas*-derived cephalosporinase (PDC; intrinsic AmpC).^14^ Region-specific prevalence of carbapenem resistance mechanisms should be considered when evaluating empirical treatment options. Previous studies reported that among carbapenem-resistant Enterobacterales, KPC was uncommon in Middle Eastern countries, except Israel, and that carbapenem-resistant Enterobacterales commonly carry NDM and OXA-48-like carbapenemases.^6^^,^^16^^,^^17^ Carbapenemase-producing Enterobacterales in Saudi Arabia have been mainly associated with acquisition of NDM and OXA-48-like carbapenemases and rarely with KPCs.^16^

In conclusion, Enterobacterales with DTR phenotypes were uncommon (1.6%–1.8% of isolates) in Middle Eastern and African countries in 2015–18 while MDR isolates were frequently identified (27.8%–36.0% of isolates). MDR *P. aeruginosa* (25.0%–36.4%) were also commonly observed. A DTR phenotype was three to four times more common among *P. aeruginosa* (5.2%–7.4%) than Enterobacterales. Ceftazidime/avibactam retained *in vitro* activity against the majority of MDR and many DTR isolates of Enterobacterales and *P. aeruginosa*. Ceftazidime/avibactam is an important treatment option for infections caused by resistant GNB that do not carry metallo-β-lactamases, particularly Enterobacterales. Increases in infections caused by DTR isolates of GNB will pose major treatment challenges.

## Supplementary Material

dlab067_Supplementary_Data

## References

[1] Magiorakos AP, Srinivasan A, Carey RB et al Multidrug-resistant, extensively drug-resistant and pandrug-resistant bacteria: an international expert proposal for interim standard definitions for acquired resistance. Clin Microbiol Infect 2012; 18: 268–81. 21793988 10.1111/j.1469-0691.2011.03570.x

[2] Kadri SS, Adjemian J, Lai YL et al Difficult-to-treat resistance in Gram-negative bacteremia at 173 US hospitals: retrospective cohort analysis of prevalence, predictors, and outcome of resistance to all first-line agents. Clin Infect Dis 2018; 67: 1803–14. 30052813 10.1093/cid/ciy378 PMC6260171

[3] Spiliopoulou I, Kazmierczak K, Stone GG. *In vitro* activity of ceftazidime/avibactam against isolates of carbapenem-non-susceptible Enterobacteriaceae collected during the INFORM global surveillance programme (2015–17). J Antimicrob Chemother 2020; 75: 384–91. 31742604 10.1093/jac/dkz456 PMC6966093

[4] Hackel M, Kazmierczak KM, Hoban DJ et al Assessment of the *in vitro* activity of ceftazidime-avibactam against multidrug-resistant *Klebsiella* spp. collected in the INFORM global surveillance study, 2012 to 2014. Antimicrob Agents Chemother 2016; 60: 4677–83. 27216054 10.1128/AAC.02841-15 PMC4958227

[5] Algwizani A, Alzunitan M, Alharbi A et al Experience with ceftazidime-avibactam treatment in a tertiary care center in Saudi Arabia. J Infect Public Health 2018; 11: 793–5. 29706317 10.1016/j.jiph.2018.04.013

[6] Alghoribi MF, Binkhamis K, Alswaji AA et al Genomic analysis of the first KPC-producing *Klebsiella pneumoniae* isolated from a patient in Riyadh: a new public health concern in Saudi Arabia. J Infect Public Health 2020; 13: 647–50. 32067931 10.1016/j.jiph.2020.01.003

[7] CLSI. *Methods for Dilution Antimicrobial Susceptibility Tests for Bacteria that Grow Aerobically—Eleventh Edition: M07-A11*. 2018.

[8] CLSI. *Performance Standards for Antimicrobial Susceptibility Testing—Thirtieth Edition: M100*. 2020.

[9] EUCAST. Breakpoint Tables for Interpretation of MICs and Zone Diameters–Version 10.0. January 2020. http://www.eucast.org/clinical_breakpoints/.

[10] Karlowsky JA, Lob SH, Raddatz J et al *In vitro* activity of imipenem/relebactam and ceftolozane/tazobactam against clinical isolates of Gram-negative bacilli with difficult-to-treat resistance and multidrug-resistant phenotypes – SMART United States 2015-2017. Clin Infect Dis 2020; doi:10.1093/cid/ciaa381. 10.1093/cid/ciaa381 32246147

[11] Giannelli M, Bussini L, Pascale R et al Prognostic utility of the new definition of difficult-to-treat resistance among patients with Gram-negative bloodstream infections. Open Forum Infect Dis 2019; 6: ofz505. 31858018 10.1093/ofid/ofz505 PMC6916520

[12] Huh K, Chung DR, Ha YE et al Impact of difficult-to-treat resistance in Gram-negative bacteremia on mortality: retrospective analysis of nationwide surveillance data. Clin Infect Dis 2020; 71: e487–e496. 31994704 10.1093/cid/ciaa084

[13] Karlowsky JA, Biedenbach DJ, Kazmierczak KM et al Activity of ceftazidime-avibactam against extended-spectrum- and AmpC β-lactamase-producing *Enterobacteriaceae* collected in the INFORM global surveillance study from 2012 to 2014. Antimicrob Agents Chemother 2016; 60: 2849–57. 26926635 10.1128/AAC.02286-15 PMC4862475

[14] Nichols WW, de Jonge BLM, Kazmierczak KM et al *In vitro* susceptibility of global surveillance isolates of *Pseudomonas aeruginosa* to ceftazidime-avibactam (INFORM 2012 to 2014). Antimicrob Agents Chemother 2016; 60: 4743–9. 27216074 10.1128/AAC.00220-16 PMC4958170

[15] de Jonge BLM, Karlowsky JA, Kazmierczak KM et al *In vitro* susceptibility to ceftazidime-avibactam of carbapenem-nonsusceptible Enterobacteriaceae isolates collected during the INFORM global surveillance study. Antimicrob Agents Chemother 2016; 60: 3163–9. 26926648 10.1128/AAC.03042-15 PMC4862516

[16] Moghnieh RA, Kanafani ZA, Tabaja HZ et al Epidemiology of common resistant bacterial pathogens in the countries of the Arab League. Lancet Infect Dis 2018; 18: e379-94. 30292478 10.1016/S1473-3099(18)30414-6

[17] Kazmierczak K, Stone G, Johnson A et al Trending analysis of ESBL screen-positive and carbapenem-non-susceptible *Enterobacteriaceae* collected in the Middle East/Africa region: ATLAS global surveillance, 2014-2017. *Thirty-first ICC and Fourth GCCMID, Dubai, United Arab Emirates*, 2019. PP45.

