# Supplementary material for: In vitro activity of ceftazidime/avibactam against clinical isolates of Enterobacterales and Pseudomonas aeruginosa from Middle Eastern and African countries: ATLAS global surveillance programme 2015–18
Source: JAC Antimicrob Resist. 2021 Jun 1;3(2):dlab067. doi: 10.1093/jacamr/dlab067 (PMC8251253; doi:10.1093/jacamr/dlab067)
Supplement: dlab067_Supplementary_Data [file dlab067_Supplementary_Data.docx]

**Supplementary data**

**Table S1.** Summary of Gram-negative isolates tested stratified by geographic region and patient location

|  |  | Number of isolates (% of isolates by geographic region and patient location) | | | | |
| --- | --- | --- | --- | --- | --- | --- |
|  | Total number of isolates | Middle East | |  | Africa | |
| Gram-negative species |  | ICU | Non-ICU |  | ICU | Non-ICU |
| *Citrobacter* spp. (all) | 334 (7.2) | 28 (5.2) | 211 (9.5) |  | 17 (3.7) | 78 (5.6) |
| *Citrobacter freundii* | 159 (3.5) | 16 (3.0) | 101 (4.6) |  | 5 (1.1) | 37 (2.7) |
| *Citrobacter koseri* | 146 (3.2) | 12 (2.2) | 92 (4.1) |  | 11 (2.4) | 31 (2.2) |
| *Enterobacter* spp. (all) | 473 (10.3) | 83 (15.4) | 167 (7.5) |  | 58 (12.7) | 165 (11.8) |
| *Enterobacter cloacae* | 407 (8.8) | 67 (12.4) | 153 (6.9) |  | 52 (11.4) | 135 (9.7) |
| *Escherichia coli* | 1345 (29.2) | 101 (18.7) | 669 (30.2) |  | 103 (22.5) | 472 (33.9) |
| *Klebsiella aerogenes* | 180 (3.9) | 37 (6.9) | 93 (4.2) |  | 19 (4.1) | 31 (2.2) |
| *Klebsiella oxytoca* | 186 (4.0) | 46 (8.5) | 84 (3.8) |  | 14 (3.1) | 42 (3.0) |
| *Klebsiella pneumoniae* | 1341 (29.1) | 174 (32.3) | 620 (28.0) |  | 174 (38.0) | 373 (26.8) |
| *Klebsiella variicola* | 37 (0.8) | 2 (0.4) | 5 (0.2) |  | 13 (2.8) | 17 (1.2) |
| *Morganella morganii* | 125 (2.7) | 8 (1.5) | 68 (3.1) |  | 14 (3.1) | 35 (2.5) |
| *Proteus* spp. (all) | 325 (7.1) | 13 (2.4) | 186 (8.4) |  | 15 (3.3) | 111 (8.0) |
| *Proteus mirabilis* | 214 (4.6) | 11 (2.0) | 116 (5.2) |  | 12 (2.6) | 75 (5.4) |
| *Proteus vulgaris* | 87 (1.9) | 0 (0) | 56 (2.5) |  | 3 (0.7) | 28 (2.0) |
| *Providencia* spp. | 108 (2.3) | 10 (1.9) | 61 (2.8) |  | 9 (2.0) | 28 (2.0) |
| *Serratia marcescens* | 147 (3.2) | 36 (6.7) | 51 (2.3) |  | 21 (4.6) | 39 (2.8) |
| Other spp. | 7 (0.2) | 1 (0.2) | 3 (0.1) |  | 1 (0.2) | 2 (0.1) |
| All Enterobacterales | 4608 (100) | 539 (100) | 2218 (100) |  | 458 (100) | 1393 (100) |
|  |  |  |  |  |  |  |
| *Pseudomonas aeruginosa* | 1358 (100) | 198 (100) | 629 (100) |  | 176 (100) | 355 (100) |

**Table S2.** Definitions of MDR and DTR used in this study

|  |  | Antimicrobial categories included in the definition of MDR^a^ and DTR^b^ | |
| --- | --- | --- | --- |
| Gram-negative bacilli  Antimicrobial category | Antimicrobial agents tested | MDR  Nonsusceptible to ≥1 agent in ≥3 antimicrobial categories | DTR  Nonsusceptible to all agents in all β-lactam categories and to fluoroquinolones |
| Enterobacterales |  |  |  |
| Extended-spectrum cephalosporins | Cefepime, ceftazidime, ceftriaxone | √ | √ |
| Carbapenems | Imipenem, meropenem | √ | √ |
| Penicillins + β-lactamase inhibitors | Piperacillin/tazobactam | √ | √ |
| Monobactams | Aztreonam | √ | √ |
| Fluoroquinolones | Levofloxacin | √ | √ |
| Aminoglycosides | Amikacin | √ |  |
| Polymyxins | Colistin | √ |  |
|  |  |  |  |
| *Pseudomonas aeruginosa* |  |  |  |
| Extended-spectrum cephalosporins | Cefepime, ceftazidime | √ | √ |
| Carbapenems | Imipenem, meropenem | √ | √ |
| Penicillins + β-lactamase inhibitors | Piperacillin/tazobactam | √ | √ |
| Monobactams | Aztreonam | √ | √ |
| Fluoroquinolones | Levofloxacin | √ | √ |
| Aminoglycosides | Amikacin | √ |  |
| Polymyxins | Colistin | √ |  |

^a^ MDR, multidrug-resistant, Magiorakos *et al*.^12^

^b^ DTR, difficult-to-treat resistance, Kadri *et al*.^5^

### **Table S3.** Susceptibility of isolates of Enterobacterales and *P. aeruginosa* from Middle East and African countries to 11 antimicrobial agents with MICs interpreted by CLSI breakpoints and stratified by patient location (ICU and non-ICU)

| Geographic region  Bacterial group/species^c^ |  |  | % Susceptible^a,b^ | | | | | | | | | | |
| --- | --- | --- | --- | --- | --- | --- | --- | --- | --- | --- | --- | --- | --- |
|  | Patient location | *n* | AMK | ATM | CAZ | CZA | COL | CRO^d^ | FEP | IPM | MEM | LVX | TZP |
| Middle East |  |  |  |  |  |  |  |  |  |  |  |  |  |
| Enterobacterales (all) | ICU | 539 | 98.9 | 70.3 | **68.1** | 99.1 | 0 | **62.2 (259)** | 73.5 | **89.4** | 97.6 | **74.2** | **83.9** |
|  | non-ICU | 2218 | 97.7 | 72.1 | **73.0** | 99.1 | 0 | **69.1 (1235)** | 72.6 | **85.3** | 98.0 | **61.9** | **88.5** |
| *Enterobacter* spp. (all) | ICU | 83 | 100 | 67.5 | 63.9 | 97.6 | 0 | 55.3 (38) | 85.5 | 84.3 | 97.6 | **86.7** | 72.3 |
|  | non-ICU | 167 | 99.4 | 73.7 | 73.1 | 97.6 | 0 | 65.8 (79) | 80.8 | 87.4 | 95.8 | **72.5** | 80.8 |
| *Enterobacter cloacae* | ICU | 67 | 100 | 62.7 | 58.2 | 97.0 | 0 | 45.2 (31) | 83.6 | 92.5 | 97.0 | 86.6 | 68.7 |
|  | non-ICU | 153 | 99.3 | 71.9 | 71.2 | 97.4 | 0 | 64.0 (75) | 79.1 | 90.8 | 95.4 | 70.6 | 79.7 |
| *Escherichia coli* | ICU | 101 | 100 | 64.4 | 65.3 | 100 | 0 | 52.8 (53) | 65.3 | 100 | 100 | **64.4** | 92.1 |
|  | non-ICU | 669 | 98.2 | 65.2 | 69.2 | 99.9 | 0 | 64.5 (363) | 64.7 | 98.8 | 99.9 | **51.9** | 91.8 |
| *Klebsiella aerogenes* | ICU | 37 | 100 | 86.5 | 75.7 | 100 | 0 | 82.4 (17) | 100 | **78.4** | 100 | 97.3 | 86.5 |
|  | non-ICU | 93 | 97.8 | 74.2 | 72.0 | 98.9 | 0 | 69.0 (42) | 93.5 | **58.1** | 97.8 | 94.6 | 78.5 |
| *Klebsiella oxytoca* | ICU | 46 | 100 | 93.5 | 97.8 | 100 | 0 | 92.3 (26) | 97.8 | 100 | 100 | 100 | 95.7 |
|  | non-ICU | 84 | 100 | 94.0 | 97.6 | 100 | 0 | 88.0 (50) | 95.2 | 100 | 100 | 89.3 | 94.0 |
| *Klebsiella pneumoniae* | ICU | 174 | 96.6 | 50.6 | 49.4 | 98.3 | 0 | **43.1 (72)** | 50.6 | 92.5 | 93.7 | 64.9 | 72.4 |
|  | non-ICU | 620 | 95.8 | 56.6 | 56.6 | 98.5 | 0 | **58.4 (351)** | 57.3 | 94.8 | 95.2 | 58.5 | 79.7 |
| *Serratia marcescens* | ICU | 36 | 100 | 97.2 | 97.2 | 100 | 0 | 92.3 (13) | 100 | 94.4 | 100 | 94.4 | 100 |
|  | non-ICU | 51 | 100 | 100 | 100 | 100 | 0 | 100 (19) | 100 | 88.2 | 100 | 94.1 | 98.0 |
| *Pseudomonas aeruginosa* | ICU | 198 | **96.5** | **62.1** | 81.8 | 93.4 | 0 | NA^e^ | 83.3 | **60.1** | **67.2** | 63.1 | 74.2 |
|  | non-ICU | 629 | **91.6** | **69.6** | 80.1 | 92.1 | 0 | NA | 80.3 | **67.9** | **74.7** | 62.6 | 75.0 |
|  |  |  |  |  |  |  |  |  |  |  |  |  |  |
| Africa |  |  |  |  |  |  |  |  |  |  |  |  |  |
| Enterobacterales (all) | ICU | 458 | 96.7 | **64.2** | **62.9** | 96.9 | 0 | **61.6 (172)** | **64.6** | 84.7 | **93.2** | 65.7 | **77.9** |
|  | non-ICU | 1393 | 98.2 | **74.0** | **74.6** | 98.3 | 0 | **75.4 (684)** | **74.3** | 85.1 | **96.6** | 68.1 | **86.4** |
| *Enterobacter* spp. (all) | ICU | 58 | 93.1 | 63.8 | 62.1 | 93.1 | 0 | 88.9 (9) | 65.5 | 77.6 | 89.7 | 75.9 | 69.0 |
|  | non-ICU | 165 | 99.4 | 71.5 | 72.7 | 98.2 | 0 | 77.8 (54) | 74.5 | 84.8 | 93.9 | 83.0 | 81.8 |
| *Enterobacter cloacae* | ICU | 52 | 92.3 | 61.5 | 59.6 | 92.3 | 0 | 87.5 (8) | 61.5 | 82.7 | 88.5 | 73.1 | 67.3 |
|  | non-ICU | 135 | 99.3 | 65.9 | 67.4 | 97.8 | 0 | 75.0 (48) | 70.4 | 85.2 | 92.6 | 80.0 | 77.8 |
| *Escherichia coli* | ICU | 103 | 99.0 | 75.7 | 75.7 | 100 | 0 | 74.4 (39) | 76.7 | 100 | 100 | 50.5 | 91.3 |
|  | non-ICU | 472 | 99.8 | 79.9 | 82.2 | 100 | 0 | 84.2 (228) | 79.4 | 98.9 | 99.6 | 58.7 | 93.4 |
| *Klebsiella pneumoniae* | ICU | 174 | 95.4 | **37.9** | **38.5** | 95.4 | 0 | **32.5 (77)** | **36.8** | 85.6 | 87.9 | 59.8 | **62.6** |
|  | non-ICU | 373 | 96.8 | **50.1** | **50.4** | 96.2 | 0 | **51.0 (196)** | **48.8** | 90.3 | 92.8 | 57.9 | **72.1** |
| *Pseudomonas aeruginosa* | ICU | 176 | 89.8 | 65.9 | **79.0** | 89.8 | 0 | NA | **73.9** | **60.2** | **66.5** | 65.9 | **70.5** |
|  | non-ICU | 355 | 92.4 | 71.0 | **85.9** | 94.1 | 0 | NA | **82.5** | **75.5** | **80.0** | 66.8 | **78.3** |

^a^ Abbreviations: AMK, amikacin; ATM, aztreonam; CAZ, ceftazidime; CZA, ceftazidime/avibactam; COL, colistin; CRO, ceftriaxone; FEP, cefepime; IPM, imipenem; MEM, meropenem; LVX, levofloxacin; TZP, piperacillin/tazobactam.

^b^ Statistically significant differences between ICU and non-ICU are bolded and underlined.

^c^ Species with fewer than 30 isolates are not shown individually.

^d^ CRO, ceftriaxone is shown with the number of isolates of Enterobacterales tested in brackets (*n*) as ceftriaxone was not tested against all isolates of Enterobacterales.

^e^ NA, not available.

### **Table S4.** Susceptibility of isolates of Enterobacterales and *P. aeruginosa* from Middle East and African countries to 11 antimicrobial agents with MICs interpreted by EUCAST breakpoints and stratified by patient location (ICU and non-ICU)

| Geographic region  Bacterial group/species^d^ |  |  | % Susceptible^a,b,c^ | | | | | | | | | | |
| --- | --- | --- | --- | --- | --- | --- | --- | --- | --- | --- | --- | --- | --- |
|  | Patient location | *n* | AMK | ATM | CAZ | CZA | COL | CRO^e^ | FEP | IPM | MEM | LVX | TZP |
| Middle East |  |  |  |  |  |  |  |  |  |  |  |  |  |
| Enterobacterales | ICU | 539 | 96.7 | 65.1 | 64.9 | 99.1 | 83.9 | **62.2 (259)** | 71.1 | 96.3 | **74.4** | 97.8 | 79.0 |
|  | non-ICU | 2218 | 95.1 | 68.3 | 68.3 | 99.1 | 81.9 | **69.1 (1235)** | 70.9 | 95.1 | **61.9** | 98.2 | 82.4 |
| *Enterobacter* spp. (all) | ICU | 83 | 100 | 60.2 | 61.4 | 97.6 | 81.9 | 55.3 (38) | 77.1 | 96.4 | **86.7** | 97.6 | 66.3 |
|  | non-ICU | 167 | 97.6 | 71.3 | 70.1 | 97.6 | 87.4 | 65.8 (79) | 77.8 | 95.8 | **72.5** | 95.8 | 76.0 |
| *Enterobacter cloacae* | ICU | 67 | 100 | 55.2 | 55.2 | 97.0 | 91.0 | **45.2 (31)** | 73.1 | 95.5 | 86.6 | 97.0 | 61.2 |
|  | non-ICU | 153 | 97.4 | 69.9 | 68.6 | 97.4 | 90.8 | **64.0 (75)** | 75.8 | 95.4 | 70.6 | 95.4 | 74.5 |
| *Escherichia coli* | ICU | 101 | 96.0 | 58.4 | 58.4 | 100 | 100 | 52.8 (53) | 62.4 | 100 | **64.4** | 100 | 91.1 |
|  | non-ICU | 669 | 94.2 | 60.7 | 61.7 | 99.9 | 99.1 | 64.5 (363) | 62.5 | 99.6 | **51.9** | 99.9 | 85.9 |
| *Klebsiella aerogenes* | ICU | 37 | 100 | 75.7 | 75.7 | 100 | 100 | 82.4 (17) | 100 | 100 | 97.3 | 100 | 81.1 |
|  | non-ICU | 93 | 96.8 | 71.0 | 69.9 | 98.9 | 100 | 69.0 (42) | 91.4 | 97.8 | 94.6 | 97.8 | 72.0 |
| *Klebsiella oxytoca* | ICU | 46 | 100 | 89.1 | 97.8 | 100 | 100 | 92.3 (26) | 97.8 | 100 | 100 | 100 | 95.7 |
|  | non-ICU | 84 | 100 | 90.5 | 96.4 | 100 | 98.8 | 88.0 (50) | 94.0 | 100 | 89.3 | 100 | 92.9 |
| *Klebsiella pneumoniae* | ICU | 174 | 92.5 | 48.3 | 48.3 | 98.3 | 97.1 | **43.1 (72)** | 49.4 | 94.3 | 64.9 | 94.3 | 63.8 |
|  | non-ICU | 620 | 93.4 | 54.4 | 54.0 | 98.5 | 98.4 | **58.4 (351)** | 55.8 | 96.1 | 58.5 | 95.6 | 70.0 |
| *Serratia marcescens* | ICU | 36 | 100 | 97.2 | 97.2 | 100 | 0 | 92.3 (13) | 97.2 | 97.2 | 94.4 | 100 | 100 |
|  | non-ICU | 51 | 98.0 | 98.0 | 100 | 100 | 7.8 | 100 (19) | 100 | 98.0 | 94.1 | 100 | 94.1 |
| *Pseudomonas aeruginosa* | ICU | 198 | **96.5** | **73.7** | 81.8 | 93.4 | 100 | NA^f^ | 83.3 | **67.7** | 63.1 | **67.2** | 74.2 |
|  | non-ICU | 629 | **91.6** | **81.6** | 80.1 | 92.1 | 99.4 | NA | 80.3 | **74.7** | 62.6 | **74.7** | 75.0 |
|  |  |  |  |  |  |  |  |  |  |  |  |  |  |
| Africa |  |  |  |  |  |  |  |  |  |  |  |  |  |
| Enterobacterales | ICU | 458 | 95.2 | **62.0** | **60.9** | 96.9 | 86.5 | **61.6 (172)** | **63.5** | 91.5 | 65.7 | 95.0 | **72.1** |
|  | non-ICU | 1393 | 96.7 | **72.0** | **71.6** | 98.3 | 83.3 | **75.4 (684)** | **73.5** | 93.7 | 68.1 | 97.6 | **81.8** |
| *Enterobacter* spp. (all) | ICU | 58 | 93.1 | 62.1 | 60.3 | 93.1 | 96.6 | 88.9 (9) | 63.8 | 86.2 | 75.9 | 93.1 | **65.5** |
|  | non-ICU | 165 | 97.6 | 69.7 | 67.9 | 98.2 | 90.3 | 77.8 (54) | 72.7 | 93.9 | 83.0 | 95.8 | **80.6** |
| *Enterobacter cloacae* | ICU | 52 | 92.3 | 59.6 | 57.7 | 92.3 | 98.1 | 87.5 (8) | 59.6 | 84.6 | 73.1 | 92.3 | 63.5 |
|  | non-ICU | 135 | 97.0 | 65.2 | 63.0 | 97.8 | 94.8 | 75.0 (48) | 68.1 | 92.6 | 80.0 | 94.8 | 76.3 |
| *Escherichia coli* | ICU | 103 | 95.1 | 71.8 | 73.8 | 100 | 99.0 | 74.4 (39) | 74.8 | 100 | 50.5 | 100 | 86.4 |
|  | non-ICU | 472 | 97.9 | 78.2 | 78.6 | 100 | 99.8 | 84.2 (228) | 79.0 | 99.6 | 58.7 | 100 | 91.1 |
| *Klebsiella pneumoniae* | ICU | 174 | 94.8 | **35.6** | **35.6** | 95.4 | 100 | **32.5 (77)** | **35.6** | 90.2 | 59.8 | 90.8 | 52.3 |
|  | non-ICU | 373 | 95.7 | **48.0** | **48.3** | 96.2 | 99.2 | **51.0 (196)** | **48.5** | 94.1 | 57.9 | 94.4 | 60.6 |
| *Pseudomonas aeruginosa* | ICU | 176 | 89.8 | 85.2 | **79.0** | 89.8 | 99.4 | NA | **73.9** | **64.8** | 65.9 | **66.5** | **70.5** |
|  | non-ICU | 355 | 92.4 | 86.2 | **85.9** | 94.1 | 100 | NA | **82.5** | **81.1** | 66.8 | **80.0** | **78.3** |

^a^ Abbreviations: AMK, amikacin; ATM, aztreonam; CAZ, ceftazidime; CZA, ceftazidime/avibactam; COL, colistin; CRO, ceftriaxone; FEP, cefepime; IPM, imipenem; MEM, meropenem; LVX, levofloxacin; TZP, piperacillin/tazobactam.

^b^ Isolates of Enterobacterales (specifically, *Morganella morganii*, *Proteus* spp., and *Providencia* spp. tested against imipenem) and *P. aeruginosa* (tested against aztreonam, ceftazidime, cefepime, imipenem, levofloxacin, and piperacillin/tazobactam) interpreted as susceptible by EUCAST MIC breakpoints included isolates testing in the susceptible, increased exposure category.

^c^ Statistically significant differences between ICU and non-ICU are bolded and underlined.

^d^ Species with fewer than 30 isolates are not shown individually.

^e^ CRO, ceftriaxone is shown with the number of isolates of Enterobacterales tested in brackets (*n*) as ceftriaxone was not tested against all isolates of Enterobacterales.

^f^ NA, not available.

**Figure S1.** Prevalence of MDR and DTR isolates, by Gram-negative species, defined by interpreting MICs using CLSI (A) and EUCAST (B) breakpoints. Only species of Enterobacterales for which DTR isolates were identified are shown individually. There were no isolates of *Providencia stuartii* with a DTR phenotype using CLSI breakpoints.


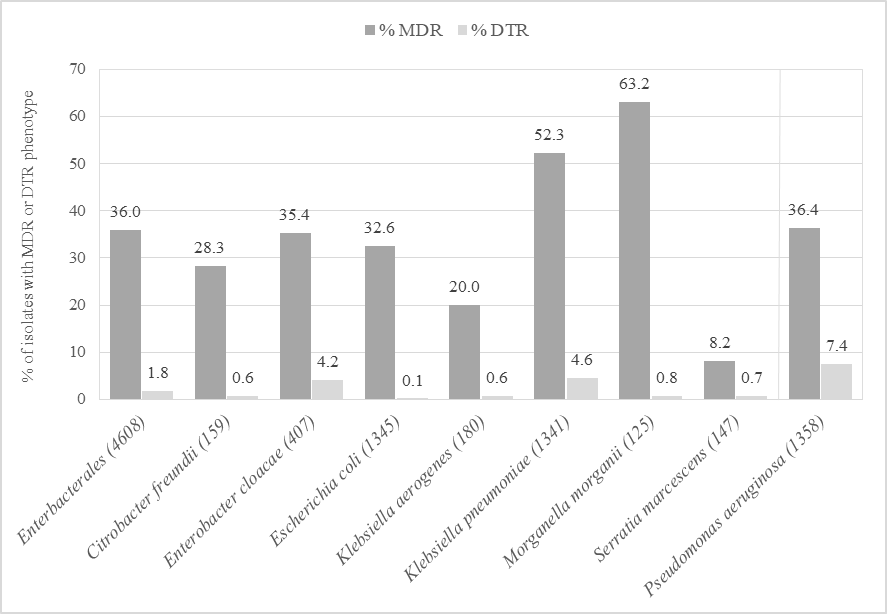


A


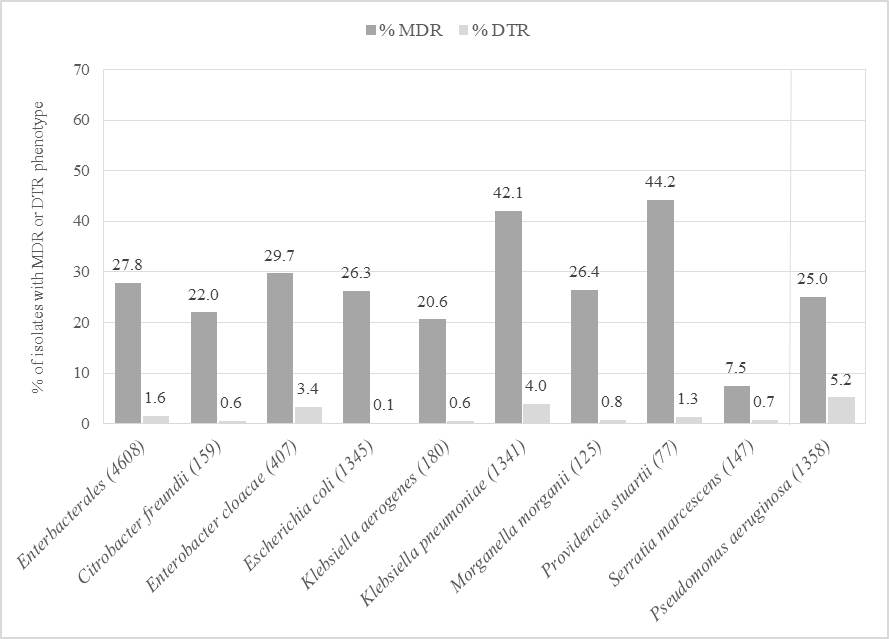


B

**Figure S2.** Prevalence of MDR and DTR isolates of Enterobacterales and *P. aeruginosa* by ward type (ICU, intensive-care unit; non-ICU, non-intensive care unit ward), defined by interpreting MICs using CLSI (A) and EUCAST (B) breakpoints.

A

B

**Figure S3.** Prevalence of MDR and DTR isolates of Enterobacterales and *P. aeruginosa* by specimen source(IAI, intraabdominal infection; RTI, respiratory tract infection; UTI, urinary tract infection; SSTI, skin and soft tissue infection; BSI, bloodstream infection) defined by interpreting MICs using CLSI (A) and EUCAST (B) breakpoints.

A

B
